# Discovery and molecular identification of a new orthophasmavirus in the Asian citrus psyllid (*Diaphorina citri*)

**DOI:** 10.3389/fmicb.2025.1570937

**Published:** 2025-07-15

**Authors:** Song Zhang, Jing Chen, Luqin Liu, Zhipeng Xie, Jiamei Liang, Fulin Yan, Yaqin Song, Xuefeng Wang, Changyong Zhou, Mengji Cao, Jinxiang Liu

**Affiliations:** ^1^Citrus Research Institute, Integrative Science Center of Germplasm Creation in Western China (CHONGQING) Science City, Southwest University, Chongqing, China; ^2^Citrus Research Institute, National Citrus Engineering and Technology Research Center, Southwest University, Chongqing, China; ^3^Guangxi Citrus Breeding and Cultivation Research Center of Engineering Technology, Guangxi Academy of Specialty Crops, Guilin, China

**Keywords:** *Bunyaviricetes*, insect viruses, RNA sequencing (RNA-seq), citrus greening disease, virome, phylogenetic analysis, virus taxonomy, virus transmission

## Abstract

**Introduction:**

Asian citrus psyllid (ACP, *Diaphorina citri*) is the primary vector of *Candidatus* Liberibacter asiaticus (*C*Las), a major causal pathogen of citrus Huanglongbing (HLB). HLB remains difficult to control, and exploring viral genomic resources may offer new strategies for managing both *C*Las and ACPs—either by utilizing viruses as biocontrol agents or as specific delivery vectors.

**Methods:**

High-throughput sequencing was employed to identify viruses associated with ACPs. A new negative-sense RNA virus, tentatively named Diaphorina citri bunyavirus 2 (DcBV2), was discovered and subsequently characterized. The quantitative distribution of DcBV2 was assessed across various developmental stages of ACPs and in different tissues of adult individuals. To investigate viral transmission patterns, progeny were obtained from mated infected ACP parents and used to feed on host plants. A potential antagonistic interaction between DcBV2 and *C*Las was explored.

**Results:**

The complete genome of DcBV2 is 11,690 nucleotides (nt) in length, comprising three segments: large (L, 6,498 nt), medium (M, 3,341 nt), and small (S, 1,851 nt), which encode the L protein with an RNA-dependent RNA polymerase domain, a glycoprotein precursor, and a nucleoprotein, respectively. DcBV2 and another ACP-associated virus, DcBV, are phylogenetically closely related; however, their L proteins shared only 54.59% amino acid sequence identity, indicating that DcBV2 is distinct. DcBV2 was detected in ACP eggs and exhibited higher titers in the salivary glands and guts of adult ACPs. It was also detected in progeny of infected ACPs and in the leaves fed upon by infected individuals. The infection rate of *C*Las was reduced in ACPs infected with DcBV2, and vice versa.

**Discussion:**

DcBV2 was fully sequenced and represents a new *Orthophasmavirus* species. It is transmitted vertically and possibly horizontally, and appears to compete with *C*Las in ACPs. These findings lay the foundation for further exploration of DcBV2’s potential in HLB management.

## 1 Introduction

The continual discovery of novel viruses in the order *Bunyavirales* has led to its considerable expansion (Kuhn et al., 2021). The International Committee on Taxonomy of Viruses (ICTV) recently upgraded the *Bunyavirales* order to the *Bunyaviricetes* class in 2024^1^ (Kuhn et al., 2024), which comprises 2 orders, 16 families, 70 genera, and 616 species as of 2025/04/09 (see text footnote 1). Among the families, *Phasmaviridae* from the order *Elliovirales* is characterized by tri-segmented (large, medium, and small), single-stranded negative-sense RNA genomes that encode a large protein (L), a glycoprotein precursor (GPC), and a nucleoprotein (N) (Kuhn and Hughes, 2024). The 5′ and 3′ ends of the genomic segments are highly conserved at the genus level and are partially reverse complementary within each segment, facilitating the formation of a panhandle structure (Schreur et al., 2018). Phasmavirids are maintained in and/or transmitted by insects and are classified into seven genera^2^ (accessed: 2025/04/09), including *Orthophasmavirus*.

Citrus has the largest cultivation area and yield among all fruit trees and is one of the most important economic crops in the world. However, citrus cultivation is frequently impacted by various diseases. Huanglongbing (HLB), also known as citrus greening disease, has become one of the most destructive diseases of citrus globally, resulting in a 30–100% yield reduction and an estimated $150 million in fruit loss annually (Chinyukwi et al., 2023). The pathogenic phloem-colonizing bacteria causing HLB are divided into three species: *Candidatus* Liberibacter asiaticus (*C*Las), *Candidatus* Liberibacter africanus (*C*Laf), and *Candidatus* Liberibacter americanus (*C*Lam) (Garnier et al., 2000; Texeira et al., 2005). *C*Las has been extensively studied due to its wide distribution and strong pathogenicity. HLB is particularly devastating to citrus in Asia, where it is caused by *C*Las. The disease can be transmitted through grafting, *Cuscuta chinensis*, and most notably, by the Asian citrus psyllids (ACPs, *Diaphorina citri*) (Lin, 1956; Chao et al., 1979; Garnier and Bové, 1983; Inoue et al., 2009), which maintain *C*Las in a circulative manner (Ghanim et al., 2016). HLB remains challenging to control (Zhao et al., 2025), and the most effective way to prevent its spread is through the implementation of integrated disease management (Morán et al., 2023).

As the primary insect vector of *C*Las, the ACP critically exacerbates the economic damage caused by HLB (Gottwald, 2010; Zhou, 2020). In addition to the *C*Las bacteria, several other viruses are associated with ACPs, including but not limited to Diaphorina citri reovirus (DcRV), Diaphorina citri bunyavirus (DcBV), Diaphorina citri picorna-like virus (DcPLV), Diaphorina citri-associated C virus (DcACV), Diaphorina citri densovirus (DcDV), all of which have been identified by high-throughput sequencing (HTS) (Marutani-Hert et al., 2009; Matsumura et al., 2016; Nigg et al., 2016; Nouri et al., 2016a, 2016b). In our previous study, *C*Las and five ACP-associated viruses, including Diaphorina citri bunyavirus (DcBV) and DcBV2 (both with only a quite small portion of sequences obtained), were detected, with a mix infection rate of approximate 60% in adult ACPs collected from geographically distinct orchards in China (Liu et al., 2024). The acquisition or transmission of one endosymbiont in insects can be influenced by another. For instance, Nhumirim virus is an insect-specific virus that inhibits West Nile virus transmission in mosquitoes (Goenaga et al., 2015).

In this study, to clarify the genomic characteristics and taxonomic status of DcBV2, we fully sequenced the large (L), medium (M), and small (S) genomic RNA segments of DcBV2, as well as the L segment of DcBV. Phylogenetic analyses based on the amino acid (aa) sequences of these three segments support that DcBV2 represents a new species within the genus *Orthophasmavirus* of the family *Phasmaviridae*. Additionally, we investigated the tissue distribution of DcBV2 in ACPs, its transmission between individuals, and discussed the potential effect of DcBV2 on *C*Las infection.

## 2 Materials and methods

### 2.1 Asian citrus psyllids and RNA extraction

A total of 100 wild ACP adults were randomly collected, regardless of sex, from citrus orchards in Guangdong (50) and Guizhou (50) provinces of China using an aspirator (Table 1). After mixing the 100 ACPs, total RNA was extracted using TRIzol reagent (Invitrogen, Carlsbad, CA, USA) for transcriptome sequencing. Additionally, 100 eggs, 20 1st–3rd instar larvae, 20 4th–5th instar larvae, and 15 adults were randomly selected for virus quantitative detection across different developmental stages of non-wild ACPs (4 replicates; 155 × 4). Furthermore, 80 non-wild ACP adults were used for virus quantitative detection in various tissues (4 replicates; 80 × 4). Moreover, newly emerged non-wild male and female adults were paired to mate and produce progeny for viral transmission studies, and data from 32 pairs (4 sets, each with 8 pairs) were used for analysis (Table 2). The mated adult ACPs and their progeny were used for virus qualitative detection. All non-wild ACPs were reared on orange jessamine (*Murraya exotica* L.) plants and collected from the special insectary in Guangxi Academy of Specialty Crops, China. The collected ACPs were immediately placed in tubes on dry ice or liquid nitrogen for transport to the laboratory, followed by storage at −80°C for later analysis. Total RNA was isolated from either the whole body or specific tissues of each ACP as described above.

**Table 1:** Information on the Asian citrus psyllid (ACP) samples used in this study.

State	Number	Place of collection	Purpose
Adult	50	Orchards in Guangdong	Pooled for RNA sequencing
Adult	50	Orchards in Guizhou	
Adult	80 × 4^a^	Insectary in Guangxi	Dissected for tissue-specific DcBV2 detection^c^
Adult	15 × 4^a^	Insectary in Guangxi	Detected for DcBV2^c^
Egg	100 × 4^a^	Insectary in Guangxi	Detected for DcBV2^c^
1st–3rd instar nymph	20 × 4^a^	Insectary in Guangxi	Detected for DcBV2^c^
4th–5th instar nymph	20 × 4^a^	Insectary in Guangxi	Detected for DcBV2^c^
Eclosion (female)	8 × 4^b^	Insectary in Guangxi	Mated for oviposition and detected for DcBV2^d^
Eclosion (male)	8 × 4^b^	Insectary in Guangxi	
Progeny (4th–5th instar nymph or adult) from the mated adults	57 + 23 + 47 + 34	Insectary in Guangxi	Detected for DcBV2^d^

a Four biological replicates, with a pooled sample consisting of ACP individuals for each replicate.

b Four mating sets, each with 8 separate pairs of adult ACP parents.

c Relatively quantitative detection of Diaphorina citri bunyavirus 2 (DcBV2) through reverse transcription and quantitative polymerase chain reaction (RT-qPCR).

d Qualitative detection of DcBV2 using RT-PCR.

**Table 2:** Vertical transmission efficiency of DcBV2 in the Asian citrus psyllids (ACPs).

Mated pair^a^	Number of pairs	Tested progeny	Infected progeny	Percentage
♀−/♂−	8	57	0	0.00
♀+/♂−	8	23	13	56.52
♀−/♂+	8	47	14	29.79
♀+/♂+	8	34	20	58.82

a ♀ is adult female ACP, ♂ is adult male ACP, and + and − represent virus-positive and virus-free, respectively.

### 2.2 Identification of viral sequences through transcriptomic sequencing

The purity, concentration, and integrity of the extracted ACP RNAs were evaluated using a Nanodrop spectrophotometer (Thermo Fisher Scientific, Waltham, MA, USA), a Qubit3.0 fluorometer (Invitrogen, Carlsbad, CA, USA), and an Agilent2100 instrument (plant RNA Nano Chip, Agilent, Santa Clara, CA, USA). The ribosome RNAs were removed using the Ribo-Zero Magnetic Kit (Epicenter, Madison, WI, USA), and the remaining RNAs were sheared by adding fragmentation buffer. The cDNA libraries were constructed and sequenced on an Illumina Hseq platform (Mega Genomics, Beijing, China) with pair-ended (PE) reads (2 × 150 bp). Raw reads were processed to remove adaptors and low-quality reads, consequently yielding clean reads, which were directly assembled into contigs using CLC Genomics Workbench 11 (Qiagen, Boston, MA, USA). Contigs were subjected to local BLASTx analysis against the viral protein sequence database (Viruses, taxid:10239, download date is November 2023) from NCBI to identify virus-related sequences.

### 2.3 Virus genome determination

The full genome of DcBV2 was sequenced from overlapping cDNAs generated by RT-PCR performed using specific primers (Supplementary Table S1) designed using Primer Premier 5 (PRIMER Biosoft, Palo Alto, CA, USA) and Oligo 7 (Wojciech Rychlik Molecular Biology Insights, Cascade, CO, USA). The 5′ and 3′ terminal sequences were amplified by the rapid amplification of cDNA ends (RACE) technique with specific primers (Supplementary Table S2) according to the manufacturer’s protocol (SMARTer RACE 5′/3′ Kit) (Takara, Dalian, China). The PCR products of expected sizes were purified using an EasyPure^®^ Quick Gel Extraction Kit (TransGen Biotech, Beijing, China), and cloned into a pGEM^®^-T Easy Vector System (Promega, Madison, WI, USA). Three clones of each amplicon were analyzed by gel electrophoresis and sequenced fully.

### 2.4 Sequence and phylogenetic analysis

CLC Sequence Viewer 7.0 (Qiagen, Boston, MA, USA) was used to predict open reading frames (ORFs) in the viral genome and compare the 5′ and 3′ terminal sequences of the three genomic segments (L, M, and S) of DcBV2. The predicted protein sequences from viral ORFs were analyzed using the Conserved Domain Database^3^ and SMART^4^ online tools to identify conserved domains (Letunic et al., 2021; Wang et al., 2023). The amino acid sequences of RdRP, GPC, and N from DcBV2, DcBV, and representative viruses of the genera in the family *Phasmaviridae*, as well as the genus *Emaravirus* in the family *Fimoviridae* (used as an outgroup), were utilized for phylogenetic analysis. The sequences were aligned using MAFFT v7.490 with the E-INS-i strategy for RdRP or the—auto strategy for GPC and N (Katoh and Standley, 2013). Aligned data were then trimmed for gaps using trimAl 1.2rev57 with the automated1 option (Capella-Gutiérrez et al., 2009). The trimmed data were used to construct maximum-likelihood trees with IQ-TREE 1.6.12, employing the automatically selected best model and 1,000 bootstrap replicates (Nguyen et al., 2015). The trees were figured and modified using FigTree v1.4.4.^5^

### 2.5 RT-qPCR and RT-PCR assays

Adult ACPs were dissected with forceps in 0.1 M phosphate-buffered saline (PBS, pH 7.2) using a stereo microscope (Leica, Wetzlar, Germany) followed by collection of guts, salivary glands, testes, ovaries, Malpighian tubules, and remaining tissues. ACPs at each developmental stage—egg, 1st–3rd instar, 4th–5th instar, and adult—were pooled into a single sample, with four biological replicates for each stage. RNA was extracted from each tissue or each pooled sample of each stage.

Reverse transcription (RT) was performed in a total volume of 20 μL using the All-In-One 5 × RT MasterMix Kit (ABM, Chongqing, China), with a final MasterMix concentration of 1 × and 1 μg of RNA extracted from ACP samples as the template. The RT reaction was carried out with an initial 15 min at 37°C, followed by 10 min at 60°C and a final 3 min at 95°C. Quantitative PCR (qPCR) was performed on a CFX96 Touch system (Bio-Rad, Berkeley, CA, USA), according to the manufacturer’s suggestions. The primer pairs qBV2-L, qBV2-M, and qBV2-L (Supplementary Table S3) were used for quantitative detection of DcBV2. A total volume of 20 μL qPCR reaction mixture was prepared using the Blast Taq™ 2 × qPCR MasterMix Kit (ABM, Jiangsu, China), with a final MasterMix concentration of 1×, 100 ng of cDNA, and 0.5 μM of each specific primer. The qPCR program was set with an initial 3 min at 95°C for denaturation, followed by 40 cycles of 30 s at 95°C for denaturation and 1 min at 60°C for annealing/extension. The *GAPDH* and *β-actin* genes were used as internal controls in the assays. RT-PCR was conducted using a PrimeScript™ One Step RT-PCR Kit (Takara, Dalian, China) in a 10 μL reaction system consisting of 1 μL RNA template, 0.4 μL Enzyme Mix, 0.4 μL of each specific primer (Supplementary Table S1), and 1.8 μL ddH2O. RT-PCR reaction parameters were set as follows: 30 min at 50°C for reverse transcription, 3 min at 94°C for denaturation, followed by 35 cycles of 30 s at 94°C for denaturation, 30 s of annealing at the optimal temperature, and extension at 72°C for the optimal time (1 kb/min), with a final extension of 3 min at 72°C.

### 2.6 Viral transmission assays

Newly emerged male and female ACP adults were collected and placed in yarn bags, with one male and one female per bag (Table 2). Each ACP would be determined to be either infected (+) or uninfected (−) with DcBV2, resulting in four possible pairings: −/−, +/−, −/+, and +/+. The gauze bag was placed over newly sprouted orange jessamine shoots, allowing the adults to feed and mate on the same shoots. After the females laid eggs, RNA was extracted from each male and female adult individually. Progeny nymphs that hatched from the eggs were placed on new, unfed plants with fresh leaves. Once the progeny reached 5th instar nymph or adult stage, they were removed, and RNA was extracted independently. After approximately 1 month, the leaves that had been intensively fed upon, as well as those from the same plant that were not fed upon, were collected and cleaned of surface contaminants using ddH2O. RNA was then extracted from each leaf sample using the RNAiso Plus reagent (TaKaRa, Dalian, China). The RNA of ACP or leaf was detected for DCBV2 using the primer pair DcBV2-L_4 (Supplementary Table S3).

### 2.7 Statistical analyses

RT-qPCR data were obtained from four biological replicates and normalized using the *GAPDH* and *β-actin* genes. Relative viral RNA copy levels were qualified using the 2^−ΔΔCT^ method. One-way analysis of variance (ANOVA) followed by Tukey’s honest significant difference test was used for multiple comparisons, with statistical significance set at *p* < 0.05. All statistical analyses were performed using GraphPad Prism 8.0 (GraphPad, San Diego, CA, USA) and Microsoft Excel. The reduction rate of virus (DcBV2 or DcBV) or *C*Las infection was calculated based on data described in our previous study (Liu et al., 2024), using a formula that divides the difference in detection rates of one agent between ACPs infected and uninfected by another, by the detection rate of the former in ACPs uninfected by the latter.

## 3 Results

### 3.1 Virus identification

**Figure 1:**
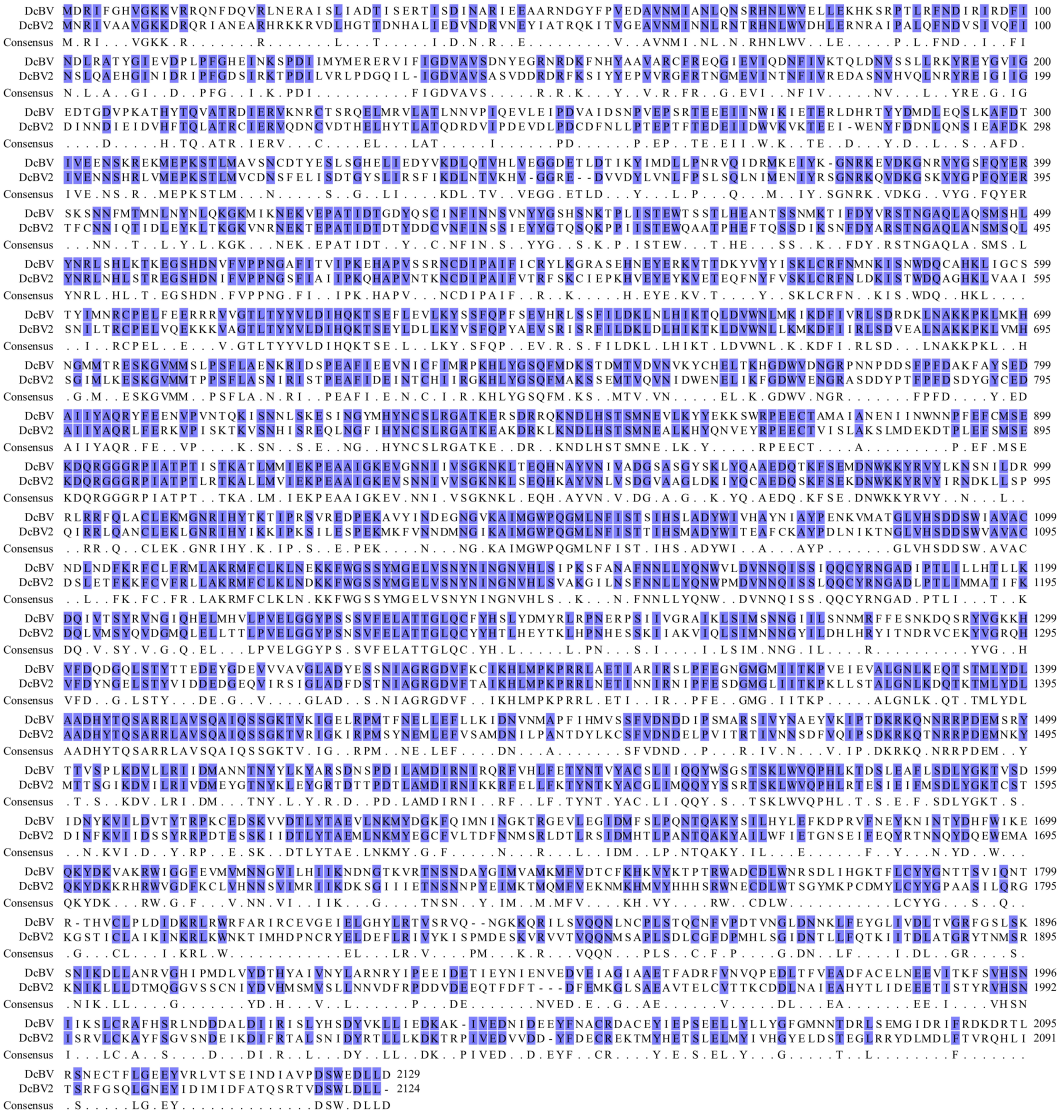
Amino acid sequence alignment of the large proteins encoded by the large genomic RNA segments of Diaphorina citri bunyavirus 2 (DcBV2) and DcBV.

A total of 36,909,278 clean reads were obtained from the pooled ACP adult sample, which comprised 100 individuals. The reads were directly assembled without removing host genome sequences to avoid excluding endogenous viral sequences, resulting in 67,047 contigs. Six contigs were identified by BLASTx analysis as phasma-related, with three contigs (lengths of 1,835–6,506 nt; sequencing depths of 583–2,077×) exhibiting 100% nucleotide (nt) sequence identity to genomic segments (L, M, and S) of Diaphorina citri bunyavirus, while the remaining three contigs (1,689–5,173 nt; 246–542×) shared only 51.11–54.66% nt sequence identity. Thus, the analyses suggested the potential presence of a new insect-infecting virus, which we have provisionally named Diaphorina citri bunyavirus 2 (DcBV2). Attempts to use untranslated sequences as signals, as previously described (Zhang et al., 2024), to explore additional contigs for either DcBV or DcBV2 in the HTS data were unsuccessful. The full-length viral genomic sequences corresponding to the three contigs of DcBV2 (NCBI GenBank accession numbers: PP025816–PP025818) and the longest contig of DcBV (PP025819) were obtained through RT-PCR, RACE, cloning, and sequencing. Sequence alignment of the largest putative proteins predicted from the longest segments of DcBV2 and DcBV revealed extensive identity in amino acid residues (Figure 1), further supporting their close relationship as indicated by the BLASTx analysis.

### 3.2 Characterization of DcBV2 genome

**Figure 2:**
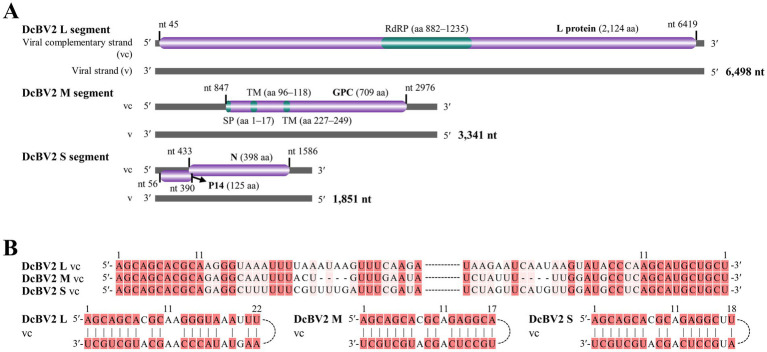
Genomic characteristics of Diaphorina citri bunyavirus 2 (DcBV2). **(A)** Genome structure of DcBV2, showing the large (L), medium (M), and small (S) RNA segments. Purple bars represent predicted open reading frames, with functional regions colored green. RdRP, RNA-dependent RNA polymerase; SP, signal peptide; TM, transmembrane regions; GPC, glycoprotein precursor; N, nucleoprotein. 5′ and 3′ refer to the 5′ and 3′ genomic ends, and nt indicate nucleotide. **(B)** Conserved and reverse complementary 5′ and 3′ ends of DcBV2 genomic segments. Identical or complementary nucleotides are highlighted with red backgrounds.

The DcBV2 genome consists of three negative-strand RNA segments (L, M, and S), with ORFs predicted on the viral complementary (positive) strand, using AUG as the start codon (Figure 2A). The ORF (nt positions 45–6,419) of the L segment (6,498 nt) encodes a 2,124-aa protein with a predicted molecular weight of 244.9 kDa. An RNA-dependent RNA polymerase (RdRP) domain (e-value: 4.49e-12) was found in the L protein at the aa positions 882–1,235. The M segment (3,341 nt) encodes (nt 847–2,976) a 709-aa putative protein with a molecular weight of 80.5 kDa, which contains a signal peptide (aa 1–17) and two transmembrane regions (aa 96–118 and 227–249). The S segment (1,851 nt) contains two ORFs, spanning nt 56–433 and 390–1,586, with a 41-nt overlap, encoding proteins with predicted molecular weights of 14.6 kDa (125 aa, P14) and 44.2 kDa (398 aa), respectively. Based on BLASTx analysis, it was concluded that the proteins predicted from L, M, and S (the larger ORF) segments of DcBV2 correspond to RdRP, glycoprotein precursor (GPC), and nucleoprotein (N), respectively. The first and last 11 nucleotides at the 5′ and 3′ ends, respectively, are identical across all three segments, and each segment contains a 17–22 nt stretch of terminal sequences that are highly reverse complementary (Figure 2B). This pattern likely facilitates the formation of a panhandle structure, a characteristic feature of conventional bunyaviruses (Guu et al., 2012). DcBV2 and its closest relative, DcBV (PP025819, KT698824 and KT698825), shared moderate sequence identities in the proteins of L (59.43% at the nt level; 54.59% at the aa level), GPC (57.16% nt; 49.16% aa), and N (57.31% nt; 50.35% aa), indicating that DcBV2 is a distinct virus.

### 3.3 Phylogenetic analysis of DcBV2

**Figure 3:**
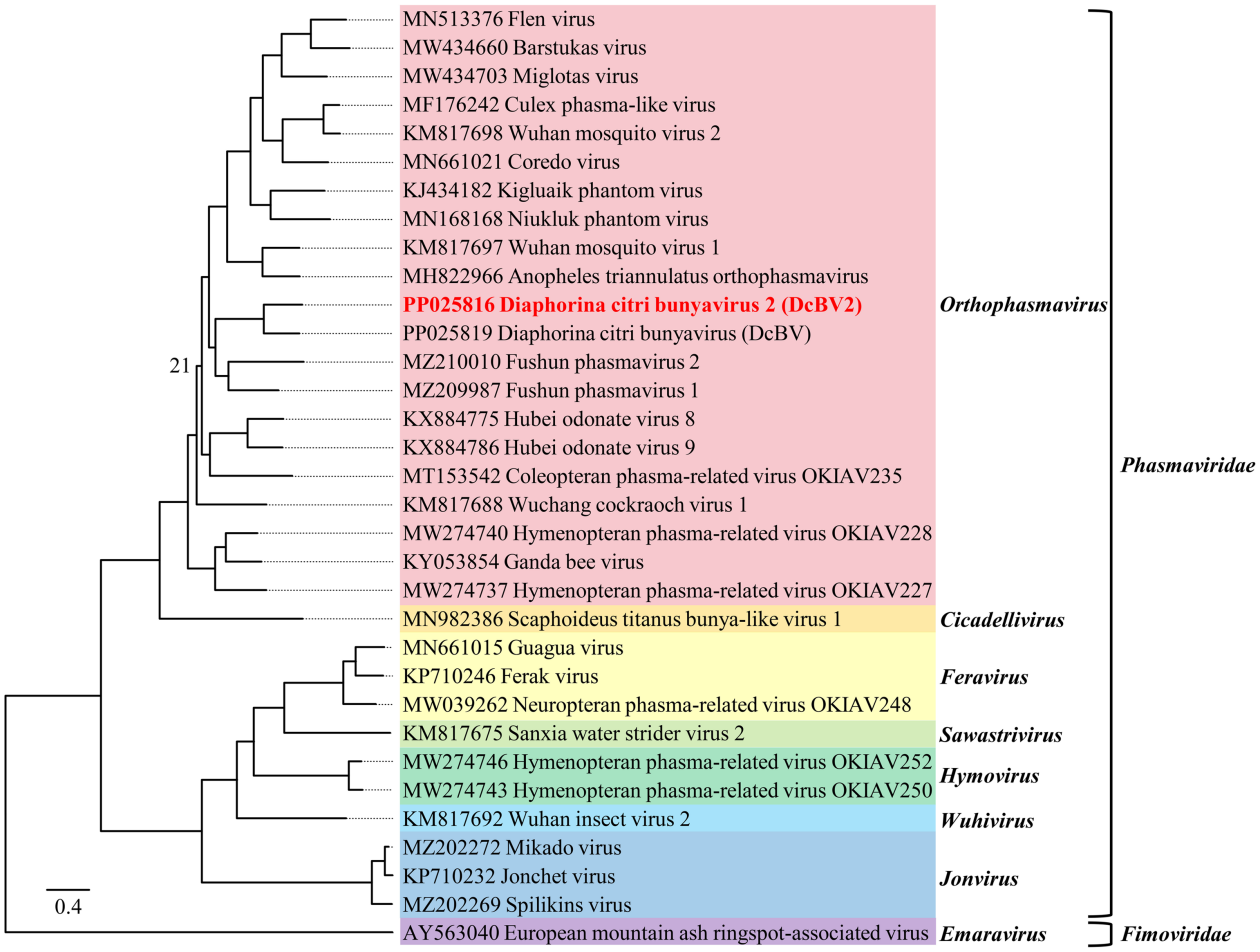
Phylogenetic analysis of the amino acid sequences of the large genomic RNA segment-encoded large polymerases from Diaphorina citri bunyavirus 2 (DcBV2), DcBV, and representative viruses from 30 species in the genera of the family *Phasmaviridae*, with European mountain ash ringspot-associated virus from the family *Fimoviridae* used as an outgroup. The maximum-likelihood method was employed using the LG+F+R5 model. Node support at the tree was determined by bootstrap analysis with 1,000 replicates, with bootstrap values shown at the nodes only if they were less than 50 (%). The scale bar represents the number of substitutions per site. Viruses from different genera are distinguished by colorful background shading. Virus names and sequence accession numbers are provided in Supplementary Table S4 in a copy-paste-friendly format.

The phylogenetic tree, constructed based on amino acid sequence alignments of the L proteins (LG+F+R5 model), showed that DcBV2 clustered with viruses in the genus *Orthophasmavirus*, family *Phasmaviridae*, particularly being closest to DcBV (Figure 3). Phylogenetic analyses of the GPC (WAG+F+R3) and N (LG+F+I+G4) amino acid sequences consistently placed DcBV2 and DcBV with other classified orthophasmaviruses (Supplementary Figure S1). Taken together with the sequence analysis, these results suggest that DcBV2 belongs to *Orthophasmavirus* within the family *Phasmaviridae*, representing a new species in the genus.

### 3.4 Distribution and transmission of DcBV2

**Figure 4:**
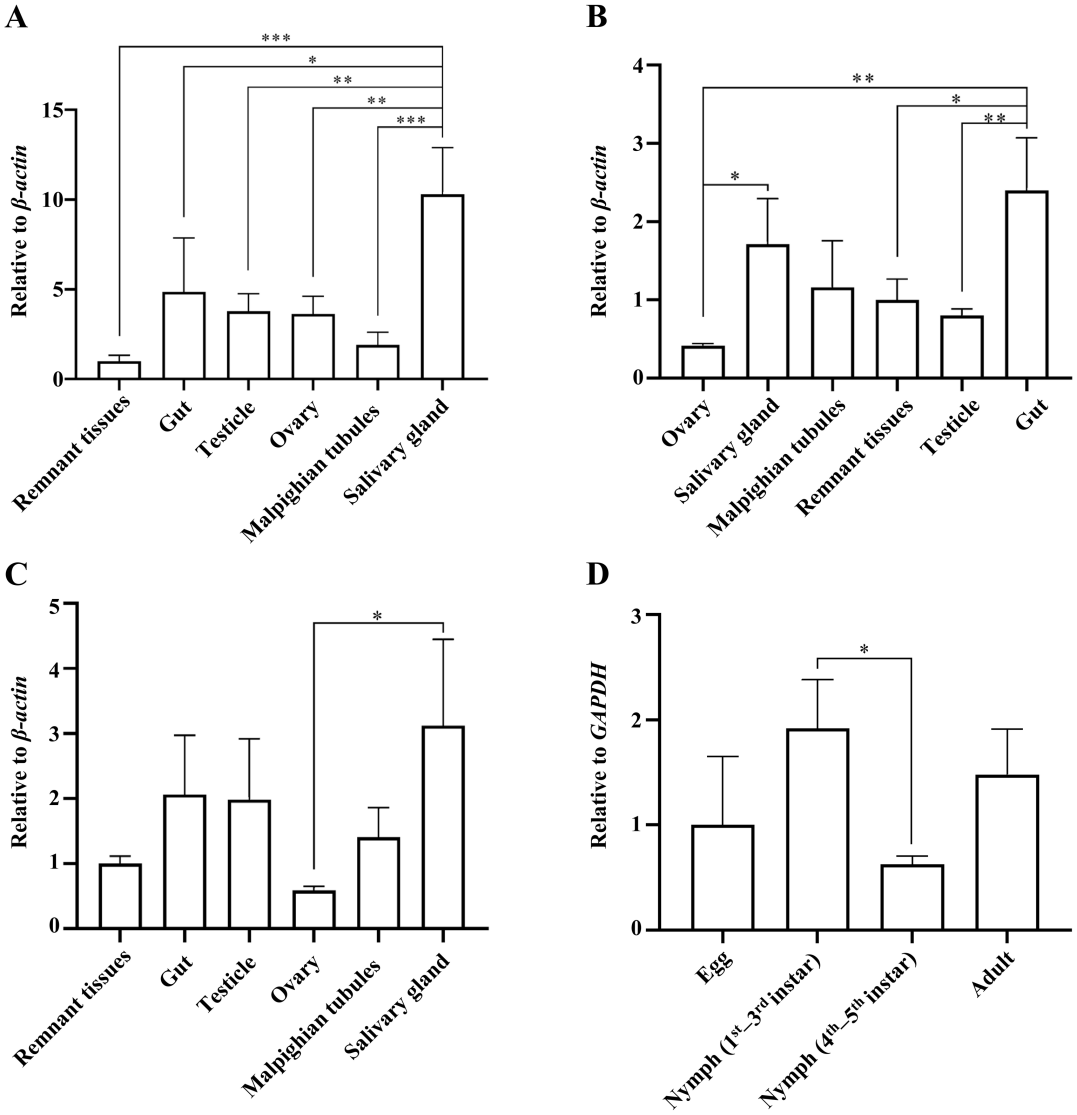
Relative titers of Diaphorina citri bunyavirus 2 (DcBV2) across different tissues and developmental stages of the Asian citrus psyllids (ACPs). Relative titers of DcBV2 in various tissues of adult ACPs, measured using the large **(A)**, medium **(B)**, and small **(C)** RNA segments, with the *β-actin* gene used for data normalization. **(D)** Relative titers of DcBV2 in various developmental stages ACPs, measured based on the large segment, with the *GAPDH* gene used for data normalization. The histograms represent means (± SE) from four biological replicates, each containing a pooled sample of ACP individuals. Statistical significance was assessed using one-way ANOVA followed by Tukey’s honest significant difference test for multiple comparisons, with * indicating significance at *p* < 0.05, ** at *p* < 0.01, and *** at *p* < 0.001.

The guts, testes, ovaries, salivary glands, Malpighian tubules, and remaining tissues of adult ACPs were dissected for quantitative analysis of DcBV2 using RT-qPCR. The results showed that the relative copy levels of the three DcBV2 RNA segments were consistently higher in the salivary glands and guts (Figures 4A–C), suggesting a tissue-specific preference that may facilitate viral horizontal transmission, if it occurs.

Eggs, 1st–3rd and 4th–5th instar larvae, and adults of ACP were tested for the presence of DcBV2. The results showed that the occurrence rate of DcBV2 was highest in the 1st–3rd instar larvae, significantly higher than in the 4th–5th instar larvae (Figure 4D), indicating variation in infection rates across developmental stages. Additionally, DcBV2 was detected in eggs, suggesting the possibility of vertical transmission. A total of 32 male–female pairs of adult ACPs were mated to generate progeny, which were tested for viral vertical transmission (Table 2). The results showed that when one of the parents was infected, the progeny were also infected, with infection rates ranging from 29.7 to 58.9%. In contrast, progeny from DcBV2-free pairs were not infected. Therefore, DcBV2 is vertically transmissible. The efficacy of vertical transmission via females was significantly higher than that via males, although infection in both parents slightly increased transmission efficacy. Moreover, weak PCR amplification bands of DcBV2 were detected in 2 of 6 orange jessamine leaf samples that had been fed on by the ACP progenies for approximately 30 days, while no bands were observed in samples that had not been fed on (Supplementary Figure S2). Thus, DcBV2 may spread horizontally through ACP feeding on plants; however, there is no evidence to suggest that DcBV2 can infect and replicate in plants.

## 4 Discussion

The widespread application of HTS techniques in viral diagnostics in recent years has improved the capacity to discover and identify novel viruses in citrus and citrus pests (Nouri et al., 2018; Chen et al., 2025; Britt-Ugartemendia et al., 2025). Accordingly, several viruses have been discovered in ACPs, which are the insect vectors that transmit *C*Las (Britt et al., 2020). In this study, two phasmavirids, DcBV and DcBV2, were identified by HTS as co-occurring in a pooled sample of adult ACPs collected from orchards in China. However, assigning HTS contigs to such co-occurring homologous segmented viruses can be challenging. A previous study obtained partial sequences of three genomic segments of DcBV, which were nearly identical to the three DcBV contigs we identified (Nouri et al., 2016b). In addition, we found that the sequencing depths of DcBV contigs were 3–8 times greater than those of the related DcBV2 contigs. Collectively, these findings allowed for the determination of the genomic RNA segments of DcBV2, suggesting a tri-segmented viral genome structure.

Three contigs of DcBV2 and the longest contig of DcBV were fully sequenced from clones of products amplified through RT-PCR and RACE experiments. Sequence analysis suggested that these correspond to the L, M, and S segments of DcBV2 and the L segment of DcBV. Like other phasmavirids, DcBV2 segments are conserved at both genomic termini, which are partially reverse complementary for each segment. The L segment of DcBV2 encodes a large protein with an RNA-dependent RNA polymerase (RdRP) domain, which is predicted to be responsible for virus replication (Walter and Barr, 2011). The M segment encodes a glycoprotein precursor (GPC), which is typically cleaved into the mature glycoproteins Gn and Gc (Schreur et al., 2018), while the S segment encodes the nucleoprotein (N). Phylogenetic analysis based on the L, GPC, and N proteins collectively supported the inclusion of DcBV2 and DcBV in the genus *Orthophasmavirus* of family *Phasmaviridae*. Since the species demarcation criteria for this genus require < 95% sequence identity in the L protein at the aa level [see website mentioned by Kuhn et al., (2024)], the 54.59% identity shared between DcBV2 and its closest relative, DcBV, suggests that DcBV2 should be classified as a member of a new species.

Quantitative analysis using RT-qPCR demonstrated that DcBV2 infects ACPs across various developmental stages, particularly eggs, indicating vertical transovarial transmission (TOT), a characteristic commonly observed among vector-borne bunyaviricetes (Bergren and Kading, 2018). Indeed, experimental evidence confirmed the TOT of DcBV2 (Table 2). Notably, infection in male ACPs alone is sufficient for DcBV2 TOT. According to the tissue-specific quantitative analysis of DcBV2 (Figure 4), it was detected, albeit at low titers, in both the ovaries (female) and testes (male) of infected adult ACPs, providing the physiological basis to support DcBV2 TOT. In contrast, DcBV2 was present in the alimentary tract (salivary gland and gut) at high loads. This distribution pattern appears conducive for horizontal oral transmission of bunyaviricetes (Horne and Vanlandingham, 2014). A similar case has been observed for Aphid bunyavirus 1 (ABV-1), which highly accumulates in the alimentary tract of aphids and may be transmitted horizontally (An et al., 2022). DcBV2 was detected in the leaves that infected ACPs had fed on (Supplementary Figure S2), suggesting that the virus is released from ACPs into plant tissues. Therefore, we do not exclude the possibility of horizontal transmission of DcBV2 among ACPs. ABV-1 has been shown to shorten developmental duration and enhance the reproductive capacity of its aphid hosts (An et al., 2022). However, it remains unclear whether DcBV2 affects the biological characteristics of ACPs.

Antagonism between microbes is ubiquitous in nature, driven by survival competition for resources and indirect effects through hosts, such as immune responses induced by the first agent (Waksman, 1941). Previous research has extensively explored the use of bacterial and viral microbes for controlling *C*Las in citrus plants (Fu et al., 2019; Poveda et al., 2021), but has focused less on *C*Las within ACPs. Our previous work showed that most ACP samples collected in the field were co-infected with multiple insect microsymbionts (Liu et al., 2024). The infection rate of *C*Las in DcBV2-infected ACPs was 17.81% lower than in DcBV2-free ACPs. Conversely, *C*Las infection also reduced DcBV2 infection by 17.3%. This reciprocal adverse effect has also been observed between *C*Las and the other four viruses, including DcBV (Liu et al., 2024). Together, these cases suggest the presence of a robust, non-specific antagonism between the viruses and *C*Las. Since *C*Las also prefers the alimentary tract for accumulation (Guo et al., 2024), DcBV2 and *C*Las may influence each other in the same ACP tissues through a currently unknown mechanism, inhibiting each other’s infection rates. However, this conjecture should be verified under strict experimental control. Additionally, to fully explore the potential of DcBV2, further studies are needed to evaluate its suitability as a viral vector.

## Data Availability

The datasets presented in this study can be found in online repositories. The names of the repository/repositories and accession number(s) can be found in the article/Supplementary material.

## Footnotes

^1^ https://ictv.global/taxonomy

^2^ https://ictv.global/report/chapter/phasmaviridae/phasmaviridae

^3^ http://www.ncbi.nlm.nih.gov/Structure/cdd/wrpsb.cgi/

^4^ https://smart.embl.de/

^5^ https://github.com/rambaut/figtree

## Supplementary material

SUPPLEMENTARY FIGURE S1 Phylogenetic analysis of the amino acid sequences of the medium genomic RNA segment-encoded glycoprotein precursors—GPC **(A)** or the small genomic RNA segment-encoded nucleoproteins—N **(B)** from Diaphorina citri bunyavirus 2 (DcBV2), DcBV, and representative viruses from 30 species in the genera of the family *Phasmaviridae*, with European mountain ash ringspot-associated virus from the family *Fimoviridae* used as an outgroup. The maximum-likelihood method was applied using the WAG+F+R3 model for GPC or the LG+F+I+G4 model for N. Node support at the tree was determined by bootstrap analysis with 1,000 replicates, with bootstrap values shown at the nodes only if they were less than 50 (%). The scale bar represents the number of substitutions per site. Viruses from different genera are distinguished by colorful background shading. Virus names and sequence accession numbers are provided in Supplementary Table S4 in a copy-paste-friendly format.

SUPPLEMENTARY FIGURE S2 Detection of Diaphorina citri bunyavirus 2 (DcBV2) in orange jessamine (*Murraya exotica* L.) leaves fed on by the Asian citrus psyllids (ACPs).
