# Supplementary material for: Discovery and molecular identification of a new orthophasmavirus in the Asian citrus psyllid (Diaphorina citri)
Source: Front Microbiol. 2025 Jul 15;16:1570937. doi: 10.3389/fmicb.2025.1570937 (PMC12303969; doi:10.3389/fmicb.2025.1570937)
Supplement: Supplementary file 3 [file Table_1.docx]

**Supplementary Tables**

**Table S1** Primers used for amplification of the full-length viral genomic RNA segments.

| **Primer name ^1^** | **Primer sequence (5ʹ-3ʹ)** | **Size (bp)** |
| --- | --- | --- |
| DcBV2-L_F1 | CATATCAGAGCGCAAGGGT | 772 |
| DcBV2-L_R1 | TCCCTATCTTGTGTTGCTAGT |  |
| DcBV2-L_F2 | CAGTAGTTCGTGGATTTAGGAC | 892 |
| DcBV2-L_R2 | CTTATTATAGGGGGCTTTTGTGAT |  |
| DcBV2-L_F3 | ATACAGAAGTGGCAACAGGAAACA | 1,135 |
| DcBV2-L_R3 | AGACTTGGCCATAAATTGACTACC |  |
| DcBV2-L_F4 | GCTGAAAGAAAGTAAAGGAGTG | 905 |
| DcBV2-L_R4 | GCAGCCTACGGATTTGAGGTG |  |
| DcBV2-L_F5 | CTTGTATCAGATGGAGTAGCAG | 1,467 |
| DcBV2-L_R5 | CATGGCAGATACGAACTCT |  |
| DcBV2-L_F6 | GTTAGCCAAGCCATACAATC | 904 |
| DcBV2-L_R6 | TGAAGTCACCAACCCATCTA |  |
| DcBV2-L_F7 | TGGGAAATGGCACAGAAAT | 1,188 |
| DcBV2-L_R7 | CCGCACTGTAAATAAGTCCATTAGAT |  |
| DcBV2-M_F1 | CTCAGCCATTATATCAAGTGCC | 1,438 |
| DcBV2-M_R1 | GTGACATCACAACAGTGTACAACG |  |
| DcBV2-M_F2 | GCAAGATGACAGGTGTCAACAC | 2,005 |
| DcBV2-M_R2 | ACCAACTGGCAATTGACATAGTAG |  |
| DcBV2-S_F1 | TACGCAGAGGCTTTTTTCG | 1,834 |
| DcBV2-S_R1 | GAGGCATCCAACATGAACTAG |  |
| DcBV-L_F1 | GAAGCAGCACGAAATGATGGA | 1,145 |
| DcBV-L_R1 | GTGGCTGGTTCAACTTTCTCA |  |
| DcBV-L_F2 | ACAAGGGAAACAGGAAGGAAG | 1,600 |
| DcBV-L_R2 | GTTGCAATTGGCCTCCCTC |  |
| DcBV-L_F3 | AATTGCTCGCTGCGGGGT | 1,477 |
| DcBV-L_R3 | CCAGCCCTACAGCGACCAC |  |
| DcBV-L_F4 | GGCAGACATACCCACGCT | 1,685 |
| DcBV-L_R4 | CCATAATTCCGTAGGCATC |  |
| DcBV-L_F5 | GAGATGGATTGGTGGCTTTG | 1,292 |
| DcBV-L_R5 | TGCCAGGATGTTGAAGAATAG |  |

Note: ^1^ L, M, and S represent the large (L), medium (M), and small (S) genomic RNA segments of Diaphorina citri bunyavirus 2 (DcBV2) or DcBV, respectively.

**Table S2** Primers used for RACE

| **Primer name** **^1^** | **Primer sequence (5ʹ-3ʹ)** |
| --- | --- |
| BV2-L_5ʹ_GSP | GATTACGCCAAGCTTGCCTTGTGGCAATATACTCATTGACACG |
| BV2-L_5ʹ_NGSP | GATTACGCCAAGCTTGCTGCCTATCCTTCTTACCTACTGCTGC |
| BV2-L_3ʹ_GSP | GATTACGCCAAGCTTGGTCTCAGTGCAGAGGCAGTTACAG |
| BV2-L_3ʹ_NGSP | GATTACGCCAAGCTTCTCAAGGACAAAACTAGGCCTATTG |
| BV2-M_5ʹ_GSP | GATTACGCCAAGCTTCAGTTTTCTGCATTAACATGTATGGCG |
| BV2-M_5ʹ_NGSP | GATTACGCCAAGCTTGAAATTGAAGTCAAACCAACACTGG |
| BV2-M_3ʹ_GSP | GATTACGCCAAGCTTAGGTGTGGGCAGCACATAGAATAG |
| BV2-M_3ʹ_NGSP | GATTACGCCAAGCTTGAGGGTGAATGGTTGTCAAGTGAC |
| BV2-S_5ʹ_GSP | GATTACGCCAAGCTTCTTCGAAGACTTCTGAAAGGGTGTG |
| BV2-S_5ʹ_NGSP | GATTACGCCAAGCTTTGGGGTAGACTTGACCACAGCCAC |
| BV2-S_3ʹ_GSP | GATTACGCCAAGCTTGATGAGGAGTCTGGGTCTGATGTGG |
| BV2-S_3ʹ_NGSP | GATTACGCCAAGCTTCGGCATGATGAGGCATCTGACATG |
| BV-L_5ʹ_GSP | GATTACGCCAAGCTTGTTCGATGGTCAAGCCTTTCAGT |
| BV-L_5ʹ_NGSP | GATTACGCCAAGCTTGGACATTGTTGAGTGTGGCAAGG |
| BV-L_3ʹ_GSP | GATTACGCCAAGCTTGCAGCAGAAACATTCGCTGACAG |
| BV-L_3ʹ_NGSP | GATTACGCCAAGCTTGACAGGACACTGAGAAGCAATGAG |
| UPM | CTAATACGACTCACTATAGGGCAAGCAGTGGTATCAACGCAGAGT |
| UPS | CTAATACGACTCACTATAGGGC |

Note: ^1^ GSP is gene specific primer, NGSP is nested GSP, UPM (Universal Primer Mix) and UPS (Universal Primer Short) are primers from SMARTer RACE 5ʹ/3ʹ Kit, and L, M, and S represent the large (L), medium (M), and small (S) genomic RNA segments of Diaphorina citri bunyavirus 2 (DcBV2, the BV2) or DcBV (the BV), respectively.

**Table S3** Primers used to quantitatively and qualitatively detect Diaphorina citri bunyavirus 2 (DcBV2) in the Asian citrus psyllids (ACPs) and used for quantitative detection of the internal control genes.

| **Primer name ^1^** | **Primer sequence (5****ʹ-3ʹ)** | **Size (bp)** |
| --- | --- | --- |
| qBV2-L_F ^2^ | TACAGAAATGGTGCTGACCTCC | 131 |
| qBV2-L_R ^2^ | CCTCCTAGTTCTACAGGCAAT |  |
| qBV2-M_F ^2^ | AAACCTATGCTGGTGTTCTCA | 133 |
| qBV2-M_R ^2^ | GCCCACACCTTAACTATTTGC |  |
| qBV2-S_F ^2^ | ACTAACTCCTCTCGCTGGTG | 80 |
| qBV2-S_R ^2^ | TGACCCTTTCATGCCCTGTC |  |
| GAPDH_F | CATGGCAAGTTCAACGGTGA | 171 |
| GAPDH_R | CGATGCCTTCTCAATGGTGG |  |
| β-actin_F | CCCTGGACTTTGAACAGGAA | 170 |
| β-actin_R | CTCGTGGATACCGCAAGATT |  |
| DcBV2-L_F4 ^3^ | GCTGAAAGAAAGTAAAGGAGTG | 905 |
| DcBV2-L_R4 ^3^ | GCAGCCTACGGATTTGAGGTG |  |

Note: ^1^ L, M, and S represent the large (L), medium (M), and small (S) genomic RNA segments of DcBV2 (the BV2), respectively; ^2^ quantitative detection of DcBV2 in various adult ACP tissues using qBV2-L, qBV2-M, and qBV2-S primer pairs, and in different ACP developmental stages using qBV2-L primer pair; ^3^ qualitative detection of DcBV2 in ACP adults using DcBV2-L_4 primer pair.

**Table S4** List of the viral genomic RNA segments used for phylogenetic tree construction.

| **Virus name ^1^** | **Accession number ^2^** | **Segment** |
| --- | --- | --- |
| Anopheles triannulatus orthophasmavirus | MH822966 | Large |
| Anopheles triannulatus orthophasmavirus | MH822967 | Medium |
| Anopheles triannulatus orthophasmavirus | MH822968 | Small |
| Barstukas virus | MW434660 | Large |
| Barstukas virus | MW434632 | Medium |
| Barstukas virus | MW434647 | Small |
| Coleopteran phasma-related virus OKIAV235 | MT153542 | Large |
| Coleopteran phasma-related virus OKIAV235 | MT153459 | Medium |
| Coleopteran phasma-related virus OKIAV235 | MT153483 | Small |
| Coredo virus | MN661021 | Large |
| Coredo virus | MN661023 | Small ? ^3^ |
| Coredo virus | MN661022 | Small |
| Culex phasma-like virus | MF176242 | Large |
| Culex phasma-like virus | MF176243 | Medium |
| Culex phasma-like virus | MF176244 | Small |
| Diaphorina citri bunyavirus (DcBV) | PP025819 | Large |
| Diaphorina citri bunyavirus (DcBV) | KT698824 | Medium |
| Diaphorina citri bunyavirus (DcBV) | KT698825 | Small |
| Diaphorina citri bunyavirus 2 (DcBV2) | PP025816 | Large |
| Diaphorina citri bunyavirus 2 (DcBV2) | PP025817 | Medium |
| Diaphorina citri bunyavirus 2 (DcBV2) | PP025818 | Small |
| European mountain ash ringspot-associated virus | AY563040 | RNA1 |
| European mountain ash ringspot-associated virus | AY563041 | RNA2 |
| European mountain ash ringspot-associated virus | DQ831831 | RNA3 |
| Ferak virus | KP710246 | Large |
| Ferak virus | KP710264 | Medium |
| Ferak virus | KP710267 | Small |
| Flen virus | MN513376 | Large |
| Flen virus | MN513375 | Medium |
| Flen virus | MN513374 | Small |
| Fushun phasmavirus 1 | MZ209987 | Large |
| Fushun phasmavirus 1 | MZ209988 | Medium |
| Fushun phasmavirus 1 | MZ209989 | Small |
| Fushun phasmavirus 2 | MZ210010 | Large |
| Fushun phasmavirus 2 | MZ210011 | Medium |
| Fushun phasmavirus 2 | MZ210012 | Small |
| Ganda bee virus | KY053854 | Large |
| Ganda bee virus | KY053855 | Medium |
| Ganda bee virus | KY053856 | Small |
| Guagua virus | MN661015 | Large |
| Guagua virus | MN661017 | Medium |
| Guagua virus | MN661016 | Small |
| Hubei odonate virus 8 | KX884775 | Large |
| Hubei odonate virus 8 | KX884776 | Medium |
| Hubei odonate virus 8 | KX884777 | Small |
| Hubei odonate virus 9 | KX884786 | Large |
| Hubei odonate virus 9 | KX884787 | Medium |
| Hubei odonate virus 9 | KX884788 | Small |
| Hymenopteran phasma-related virus OKIAV227 | MW274737 | Large |
| Hymenopteran phasma-related virus OKIAV227 | MW274738 | Medium |
| Hymenopteran phasma-related virus OKIAV227 | MW274739 | Small |
| Hymenopteran phasma-related virus OKIAV228 | MW274740 | Large |
| Hymenopteran phasma-related virus OKIAV228 | MW274741 | Medium |
| Hymenopteran phasma-related virus OKIAV228 | MW274742 | Small |
| Hymenopteran phasma-related virus OKIAV250 | MW274743 | Large |
| Hymenopteran phasma-related virus OKIAV250 | MW274744 | Medium |
| Hymenopteran phasma-related virus OKIAV250 | MW274745 | Small |
| Hymenopteran phasma-related virus OKIAV252 | MW274746 | Large |
| Hymenopteran phasma-related virus OKIAV252 | MW274747 | Medium |
| Hymenopteran phasma-related virus OKIAV252 | MW274748 | Small |
| Jonchet virus | KP710232 | Large |
| Jonchet virus | KP710240 | Medium |
| Jonchet virus | KP710243 | Small |
| Kigluaik phantom virus | KJ434182 | Large |
| Kigluaik phantom virus | KJ434183 | Medium |
| Kigluaik phantom virus | KJ434184 | Small |
| Miglotas virus | MW434703 | Large |
| Miglotas virus | MW434675 | Medium |
| Miglotas virus | MW434690 | Small |
| Mikado virus | MZ202272 | Large |
| Mikado virus | MZ202273 | Medium |
| Mikado virus | MZ202274 | Small |
| Neuropteran phasma-related virus OKIAV248 | MW039262 | Large |
| Neuropteran phasma-related virus OKIAV248 | MW039263 | Medium |
| Neuropteran phasma-related virus OKIAV248 | MW039264 | Small |
| Niukluk phantom virus | MN168168 | Large |
| Niukluk phantom virus | MN168169 | Medium |
| Niukluk phantom virus | MN168170 | Small |
| Sanxia water strider virus 2 | KM817675 | Large |
| Sanxia water strider virus 2 | KX650646 | Medium |
| Sanxia water strider virus 2 | KX650647 | Small |
| Scaphoideus titanus bunya-like virus 1 | MN982386 | Large |
| Scaphoideus titanus bunya-like virus 1 | MN982384 | Medium |
| Scaphoideus titanus bunya-like virus 1 | MN982385 | Small |
| Spilikins virus | MZ202269 | Large |
| Spilikins virus | MZ202270 | Medium |
| Spilikins virus | MZ202271 | Small |
| Wuchang cockraoch virus 1 | KM817688 | Large |
| Wuchang cockraoch virus 1 | KM817721 | Medium |
| Wuchang cockraoch virus 1 | KM817748 | Small |
| Wuhan insect virus 2 | KM817692 | Large |
| Wuhan insect virus 2 | KM817724 | Medium |
| Wuhan insect virus 2 | KM817753 | Small |
| Wuhan mosquito virus 1 | KM817697 | Large |
| Wuhan mosquito virus 1 | KM817726 | Medium |
| Wuhan mosquito virus 1 | KM817758 | Small |
| Wuhan mosquito virus 2 | KM817698 | Large |
| Wuhan mosquito virus 2 | KM817727 | Medium |
| Wuhan mosquito virus 2 | KM817759 | Small |

Note: ^1^ the list is ordered by virus names; ^2^ the corresponding genomic RNA segment sequences can be retrieved from NCBI using Batch Entrez (https://www.ncbi.nlm.nih.gov/sites/batchentrez) and accession numbers; ^3^ the medium genomic RNA segment of this virus seems to be another small segment that encodes nucleoprotein.
